# Human fluoroacetate poisoning: A case series of 36 patients in Vietnam

**DOI:** 10.1016/j.toxrep.2025.102189

**Published:** 2025-12-14

**Authors:** Nguyen Dang Duc, Lam Nguyen Hong Anh, Lam Nguyen Hong Khanh, Nguyen Dang Bach

**Affiliations:** a Poison Control Center, Bach Mai Hospital, Hanoi, Viet Nam; b Thai Nguyen University of Medicine and Pharmacy, Thai Nguyen, Viet Nam; c Vinh University of Medicine and Pharmacy, Nghe An, Viet Nam; d Hanoi Medical University, Hanoi, Viet Nam

**Keywords:** Fluoroacetate poisoning, Sodium fluoroacetate, Vietnam, Clinical features, ICU admission, Outcome

## Abstract

Fluoroacetate poisoning is a rare but potentially lethal condition. We retrospectively reviewed 36 consecutive patients (27 males and 9 females) with confirmed poisoning treated at the Poison Control Center, Bach Mai Hospital, Hanoi, Vietnam, from June 2023 to December 2024. The mean age was 35.4 ± 12.6 years. The median time from ingestion to hospital admission was 3.5 h. On admission, 69.4 % of patients were asymptomatic, 22.2 % had seizures, and 8.3 % presented with altered consciousness. The mean Glasgow Coma Scale (GCS) score was 13.9 ± 2.0 (range 5–15). Median serum creatinine and creatine kinase (CK) levels were 72 µmol/L and 155 U/L, respectively; 16.7 % of patients had CK > 1000 U/L. Median ionized calcium was 1.015 mmol/L, and 19.4 % had serum lactate ≥ 2 mmol/L. Gastric lavage and activated charcoal were administered in 44.4 % and 36.1 % of cases, respectively. Six patients (16.7 %) required intensive care unit (ICU) admission, and no deaths occurred. Overall, most patients presented early with mild manifestations and had favorable short-term outcomes under supportive management.

## Highlights

• This is the first systematic clinical case series of human fluoroacetate poisoning in Vietnam.
• Most patients (69.4 %) presented early and were asymptomatic despite confirmed exposure.
• No deaths occurred among 36 patients, contrasting with historically high fatality rates.
• A minority developed significant abnormalities, including CK > 1000 U/L in 16.7 % of cases.
• Lower GCS scores on admission were more common among patients requiring ICU care.
• Early recognition and supportive management were associated with favorable short-term outcomes.

## 1 Introduction

Fluoroacetate (commonly as sodium fluoroacetate, “1080”), a potent metabolic poison, inhibits the tricarboxylic acid cycle through aconitase blockade, resulting in cellular energy failure and severe metabolic derangements [1], [2]. It has been widely used as a rodenticide in several countries, although its use is banned or restricted in many regions because of its high toxicity to humans and animals [3], [4]. Human fluoroacetate poisoning remains uncommon but carries a high risk of mortality due to refractory seizures and cardiovascular collapse [5], [6].

Reported outbreaks have occurred in China, Brazil, and South Africa, often linked to accidental or deliberate ingestion of contaminated food or illegal pesticides [7], [8]. However, data from Southeast Asia are extremely limited, and no systematic clinical series from Vietnam has been previously reported.

Management of fluoroacetate poisoning remains largely supportive, as no specific antidote is available [9], [10]. Early decontamination and correction of metabolic abnormalities may improve outcomes, but prognostic indicators are poorly defined [11], [12].

This study describes 36 (27 males and 9 females) consecutive patients with confirmed fluoroacetate poisoning treated at a national referral toxicology center in Vietnam from June 2023 to December 2024. To our knowledge, this is the first clinical case series from Vietnam, aiming to characterize the clinical spectrum, laboratory findings, management strategies, and factors associated with intensive care unit admission [13], [14].

## 2 Methods

We conducted a retrospective case series including all consecutive patients with confirmed fluoroacetate poisoning treated at the Poison Control Center, Bach Mai Hospital, Hanoi, Vietnam, between 1 June 2023 and 31 December 2024.

Patients were eligible if they presented with a clinical picture consistent with acute fluoroacetate ingestion and had laboratory confirmation of fluoroacetate in blood specimens performed at our hospital’s toxicology laboratory. Detection was carried out using gas chromatography–mass spectrometry (GC–MS; Agilent Technologies, USA), with a lower limit of detection of 0.01 mg/L. Patients lacking sufficient clinical data for extraction of key study variables were excluded.

Clinical records, both electronic and paper-based, were reviewed using a standardized data extraction form developed for the study. Two clinicians independently extracted all data; discrepancies were resolved through consensus with a senior reviewer. The following variables were recorded: demographic characteristics; exposure details (route, suspected source, and intent); time from ingestion to hospital arrival; presenting symptoms; Glasgow Coma Scale (GCS) score on admission; vital signs; electrocardiographic findings; and initial laboratory values including serum creatinine, creatine kinase (CK), ionized calcium, and serum lactate.

Rhabdomyolysis was defined as CK > 1000 U/L and hyperlactatemia as lactate ≥ 2 mmol/L. Data on management (gastric lavage, activated charcoal, anticonvulsants, circulatory support, and extracorporeal treatments), ICU admission, hospital stay, and in-hospital outcome were also collected.

Continuous variables were summarized as mean ± standard deviation (SD) or median (interquartile range, IQR) depending on data distribution, and categorical variables as counts and percentages. Comparisons between ICU and non-ICU groups used the Mann–Whitney *U* test for continuous variables and Fisher’s exact test for categorical variables. Given the small number of ICU events (n = 6), we did not perform multivariable logistic regression because of limited statistical power and risk of model instability. Analyses were conducted using IBM SPSS Statistics version 27 (IBM Corp., USA). Missing data were handled using complete-case analysis.

The study was approved by the Institutional Review Board of Bach Mai Hospital (approval number BM-TOX/IRB/2023–07). Given the retrospective, anonymized nature of the data, the requirement for informed consent was waived.

## 3 Results

A total of 36 patients (27 males and 9 females) were included in the study. The mean age was 35.4 ± 12.6 years (median 34.5; range 16–67). The median time from ingestion to hospital admission was 3.5 h (range 1–120 h). On admission, 25/36 (69.4 %) patients were asymptomatic, 8/36 (22.2 %) presented with seizures, and 3/36 (8.3 %) had altered consciousness. No cardiac arrhythmia was recorded in any patient. The mean Glasgow Coma Scale (GCS) score on admission was 13.9 (range 5–15).

Regarding laboratory findings, the median serum creatinine was 72 µmol/L (range 43–333), and the median creatine kinase (CK) level was 155 U/L. The mean CK was markedly higher (739.3 U/L) due to a few outlier cases; 6/36 (16.7 %) patients had CK > 1000 U/L. The median serum lactate was 0.95 mmol/L (mean 1.26 mmol/L), with 7/36 (19.4 %) patients having lactate ≥ 2 mmol/L. The median ionized calcium was 1.015 mmol/L, suggesting mild hypocalcemia in some cases. Gastric lavage was performed in 16/36 (44.4 %) patients and activated charcoal in 13/36 (36.1 %) patients. Electrocardiography was performed in all cases (36/36).

Six patients (16.7 %) required ICU admission during hospitalization. The median length of hospital stay was 8 days (range 2–15). No in-hospital deaths occurred. Given the limited number of ICU cases, further inferential analysis was not performed (Table 1).

**Table 1:** Baseline characteristics and laboratory findings of 36 patients.

Variable	Value
Sex, n (%)	Male 27 (75), Female 9 (25)
Age (years), mean ± SD	35.4 ± 12.6
Time from ingestion to hospital admission (h)	Median 3.5 (range 1–120)
Clinical manifestations on admission, n (%)	
Asymptomatic Seizures Altered consciousness Cardiac arrhythmia	25 (69.4) 8 (22.2) 3 (8.3) 0
GCS score on admission, mean ± SD (range)	13.9 ± 2 (5–15)
Serum creatinine (µmol/L), median (range)	72 (43–333)
Creatine kinase (U/L), median CK > 1000 U/L, n (%)	155 (range 35–4850) 6 (16.7)
Ionized calcium (mmol/L), median	1.015 (range 0.68–1.19)
Gastric lavage, n (%)	16 (44.4)
Activated charcoal, n (%)	13 (36.1)
ECG performed, n (%)	36 (100)
ICU admission, n (%)	6 (16.7)
Length of hospital stay (days), median	8 (range 2–15)
Mortality, n (%)	0

Abbreviations: GCS = Glasgow Coma Scale; CK = creatine kinase; ECG = electrocardiogram.

## 4 Discussion

This case series describes the clinical spectrum, laboratory abnormalities, management, and short-term outcomes of 36 patients with confirmed human fluoroacetate poisoning treated at a tertiary toxicology referral center in Vietnam.

To our knowledge, this represents the first systematic clinical series from Vietnam and adds region-specific data to a literature that remains sparse [1], [2], [14].

Fluoroacetate toxicity results from conversion to fluorocitrate, which inhibits aconitase in the tricarboxylic acid cycle, leading to cellular energy failure and downstream metabolic disturbances. These mechanisms explain the prominent neurological and metabolic manifestations described in prior experimental and clinical studies, including seizures, encephalopathy, and cardiovascular instability [1], [2], [3], [7].

In our cohort, the majority of patients presented early after exposure and with mild or no symptoms. Nearly 70 % were asymptomatic on admission, the median time to hospital arrival was 3.5 h, and no fatalities occurred. These findings contrast with historical reports describing high mortality and severe cardiopulmonary complications, suggesting that early recognition and timely supportive care may contribute to improved short-term outcomes in selected patients [4], [5], [6], [13], [14].

Laboratory abnormalities were heterogeneous. Although median creatine kinase (CK) and lactate levels were relatively low, a subset of patients developed marked CK elevations (> 1000 U/L), indicating clinically relevant rhabdomyolysis. This may be related to seizures, muscle ischemia, or direct toxic effects, underscoring the importance of monitoring muscle enzymes and managing convulsions promptly [1], [2], [6].

Cardiac arrhythmias, which have been reported in fatal or severe cases in earlier literature, were not observed in this series. Differences in exposure dose, formulation, timing of presentation, or case ascertainment may partially explain this discrepancy. Importantly, the absence of fatalities in our cohort should not be interpreted as evidence of low intrinsic toxicity, as severe and fatal cases have been well documented in other settings, particularly with delayed presentation or higher-dose exposure [4], [5], [7], [13].

Management remains largely supportive, as no specific antidote for fluoroacetate poisoning is currently established. Early gastrointestinal decontamination, seizure control, correction of metabolic abnormalities, and close monitoring remain the cornerstone of care. The role of extracorporeal therapies remains uncertain and is supported only by isolated case reports [1], [2], [8], [9], [10].

This study has several limitations. It is a retrospective, single-center case series with a relatively small sample size. The limited number of ICU admissions precluded robust inferential or multivariable analyses. Long-term neurological outcomes were not systematically assessed, and delayed sequelae may therefore be underestimated [11], [12].

In conclusion, this Vietnamese case series demonstrates that early-presenting patients with fluoroacetate poisoning may experience favorable short-term outcomes with supportive care, while a minority develop significant complications requiring intensive management. Larger, multicenter studies are needed to better define prognostic factors and evaluate potential targeted therapies [1], [2], [14].

## 5 Conclusion

This first systematic Vietnamese case series demonstrates that many patients with fluoroacetate ingestion present early with mild or no symptoms and may recover with timely supportive care. However, a minority develop significant laboratory abnormalities and require intensive care. Given the toxin’s well-documented potential for severe and fatal outcomes in other contexts, continued clinical vigilance and further multicenter research are warranted.

## CRediT authorship contribution statement

**Anh Hong Nguyen Lam:** Formal analysis. **Duc Dang Nguyen:** Writing – review & editing, Writing – original draft, Supervision. **Bach Dang Nguyen:** Software. **Khanh Hong Nguyen:** Methodology.

## Declaration of Competing Interest

The authors declare the following financial interests/personal relationships which may be considered as potential competing interests: Nguyen Dang Duc reports was provided by Bach Mai Hospital. Nguyen dang duc reports a relationship with Bach Mai Hospital that includes: employment. Duc Dang Nguyen has patent N/a pending to N/a. Nguyen Dang Duc, draft and vision this manuscription Lam Nguyen Hong Anh, methods Lam Nguyen Hong Khanh, data Nguyen Dang Duc, software If there are other authors, they declare that they have no known competing financial interests or personal relationships that could have appeared to influence the work reported in this paper.

## Data Availability

The data that has been used is confidential.

## References

[1] Goncharov N.V., Savelieva E.N., Zinchenko V.V., Kuznetsov S.A., Mindukshev I.M., Vinokurov M.V. (2006). Toxicology of fluoroacetate: a review, with possible directions for therapy research. J. Appl. Toxicol..

[2] Proudfoot A.T., Bradberry S.M., Vale J.A. (2006). Sodium fluoroacetate poisoning. Toxicol. Rev..

[3] DeLey Cox V.E., Hartog M.A., Pueblo E., Racine M., Jennings L., Tressler J. (2020). Methylene blue and monosodium glutamate improve neurologic signs after fluoroacetate poisoning. Ann. N. Y. Acad. Sci..

[4] Harrisson J.W.E., Ambrus J.L., Ambrus C.M., Rees E.W., Peters R.H., Jr, Reese L.C., Baker T. (1952). Acute poisoning with sodium fluoroacetate (compound 1080). JAMA.

[5] Brockmann J.L., McDowell A.V., Leeds W.G. (1955). Fatal poisoning with sodium fluoroacetate: report of a case. JAMA.

[6] Ávila Reyes D. (2020). Sodium fluoroacetate’s poisoning: a case report. Rev. Toxicol..

[7] McCranor B.J., DeLey Cox V.E. (2019). The cardiopulmonary effects of sodium fluoroacetate. Cogent Biol..

[8] Wen W., Gao H., Kang N., Lu A., Qian C., Zhao Y. (2017). Treatment of severe fluoroacetamide poisoning in patient with combined multiple organ dysfunction syndrome by evidence-based integrated Chinese and Western medicines: a case report. Medicine.

[9] Guo Y., Qiu Y., Chen M. (2015). Toxic encephalopathy caused by fluoroacetamide with mediastinal emphysema: a case report. J. Clin. Emerg..

[10] Lu A., Yuan F., Yao Y., Wen W., Lu H., Wu S., Wang L. (2021). Reversible leukoencephalopathy caused by 2 rodenticides bromadiolone and fluoroacetamide: a case report and literature review. Medicine.

[11] Kim J.-M., Jeon B.-S. (2009). Survivors from β-fluoroethyl acetate poisoning show a selective cerebellar syndrome. J. Neurol. Neurosurg. Psychiatry.

[12] Jin J.H., Lee E.S. (2017). Isolated cerebellar atrophy due to rodenticide (β-fluoroethyl acetate) intoxication: MR findings and clinical correlation. J. Neurol. Sci. (J. Neurol. Sci.).

[13] Paradis C., Zarrouk E., de Ferrières O., Foulgoc H., Langrand J., Lan M. (2023). Fatal case of sodium fluoroacetate poisoning in two young children. Toxicol. Anal. Clin. (Toxicol. Anal. et Clin.).

[14] McTaggart D.R. (1970). Poisoning due to sodium fluoroacetate (“1080”). Med. J. Aust..

